# Data on Nitrate–Nitrite pollution in the groundwater resources a Sonqor plain in Iran

**DOI:** 10.1016/j.dib.2018.08.023

**Published:** 2018-08-13

**Authors:** Davoud Jalili, Majid RadFard, Hamed Soleimani, Samira Nabavi, Hesam Akbari, Hamed Akbari, Ali Kavosi, Abbas Abasnia, Amir Adibzadeh

**Affiliations:** a Department of Environmental Health Engineering, School of Health, Ahvaz Jundishapur University of Medical Sciences, Ahvaz, Iran; b Health deputy shahrekord University of Medical Sciences, shahrekord, Iran; c M.Sc. of Nursing, Nursing Research Center, Faculty Member Golestan University of Medical Sciences, Gorgan,Iran; d Department of Environmental Health Engineering, School of Public Health, Tehran University of Medical Sciences, Tehran, Iran; e Health Research Center, Life Style Institute, Baqiyatallah University of Medical Sciences, Tehran, Iran

**Keywords:** Nitrate and nitrite concentration, GIS, Sonqor, Groundwater resources, Iran

## Abstract

Nitrate is a groundwater pollutant which in higher concentrations limits, leads to health hazard such as Methemoglobinemia and formation of nitrosamine compounds. In this research, the nitrate and nitrite concentrations in all water resources in the villages of Songor plain were determined and the relationship between these values with the water table and zonation of nitrate concentration were investigated in the GIS environment. In this study, 37 samples of all groundwater resources of Sonqor plain were taken in, high water (March 2016) and low water (October 2017) periods. Water nitrate levels were then determined by spectrophotometry and results compared with national standards of Iran and analyzed by SPSS. Finally, the concentration distribution mapping was carried out in GIS environment and the factors affecting nitrite changes were analyzed. Nitrate concentration of water resources of Sonqor plain was fluctuating at 3.09–88.5 mg per Liter. In one station, nitrite concentrations in the high (88.5 mg/L) and low (71.4 mg/L) water seasons were higher than the maximum limit. Low thickness of alluvium, the site of wells in the downstream farmlands, the farming situation of the region, nitrate leaching from agricultural soils and wide use of nitrogen fertilizers in agriculture were considered as the causes of the pollution in one station. Though the average concentration of nitrate and nitrite are not high in this region, but because of problematic consequences of high nitrate concentrations to human health, proper management in use of chemical fertilizers, treatment or disposal of contaminated wells and protection of water wells is highly recommended.

## Specifications Table

**Table:** 

Subject area	Chemistry
More specific subject area	Water monitoring and quality
Type of data	Table, Figure
How data was acquired	To so sampling, polyethylene bottles with 1-L volume were used and samples were transferred to lab. The whole sampling steps, transferring and data analysis were conducted according to standard method of nitrite and nitrate quantification, exploiting DR5000 Spectrophotometer
Data format	Raw, analyzed
Experimental factors	The mentioned parameters above, were analyzed according to the standards for water and wastewater treatment handbook.
Experimental features	Measuring the concentration of NO^−3^ and NO^−2^ in the samples
Data source location	Sonqor, Kermanshah province, Iran
Data accessibility	The data are available with this article

## Value of the data

• Nitrate and nitrite compounds are among the contaminating factors of groundwater resources. In recent years, their average levels due to the expansion of urban, industrial and agricultural sewage and etc have increased the level of groundwater resources.
• Increasing the amount of nitrate above the permissible level can lead to health problems such as Methemoglobinemia.
• Though the average concentration of nitrate and nitrite are not high in this region, but because of problematic consequences of high nitrate concentrations to human health, proper management in use of chemical fertilizers, treatment or disposal of contaminated wells and protection of water wells are highly recommended.

## 1 Data

**Fig. 1:**
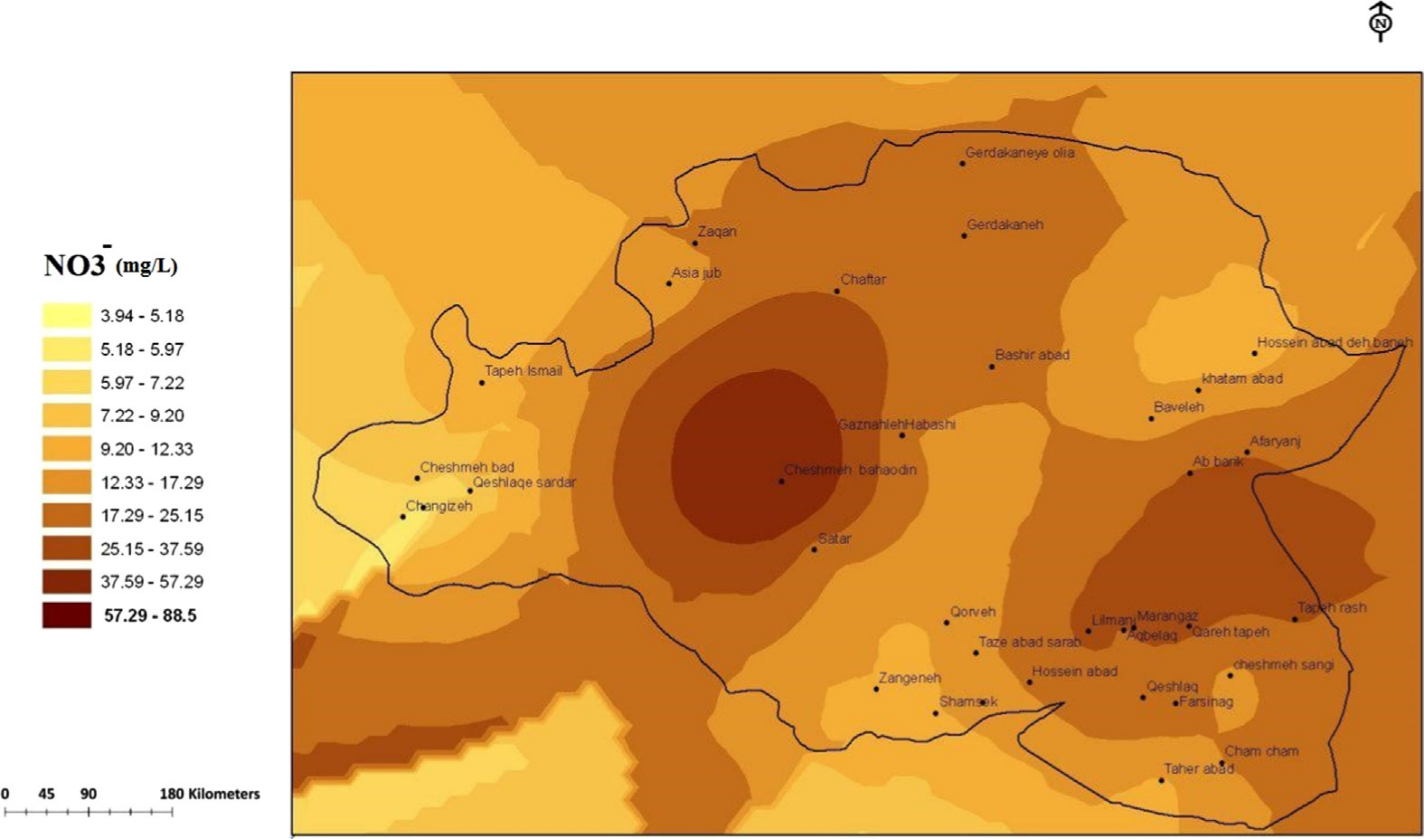
The trend of nitrate (mg/L) changes in water supply sources in the villages of the plain of Sonqor, sampling of high water season, March 2016.

**Fig. 2:**
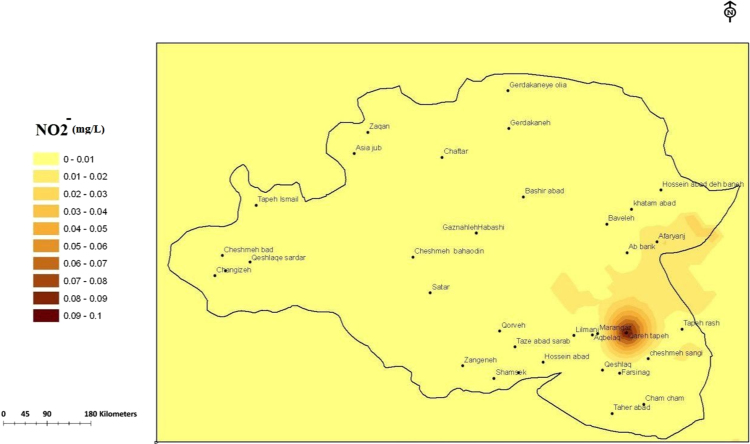
The trend of nitrite (mg/L) changes in water supply sources in the villages of the plain of Sonqor, sampling of high water season, March 2016.

**Fig. 3:**
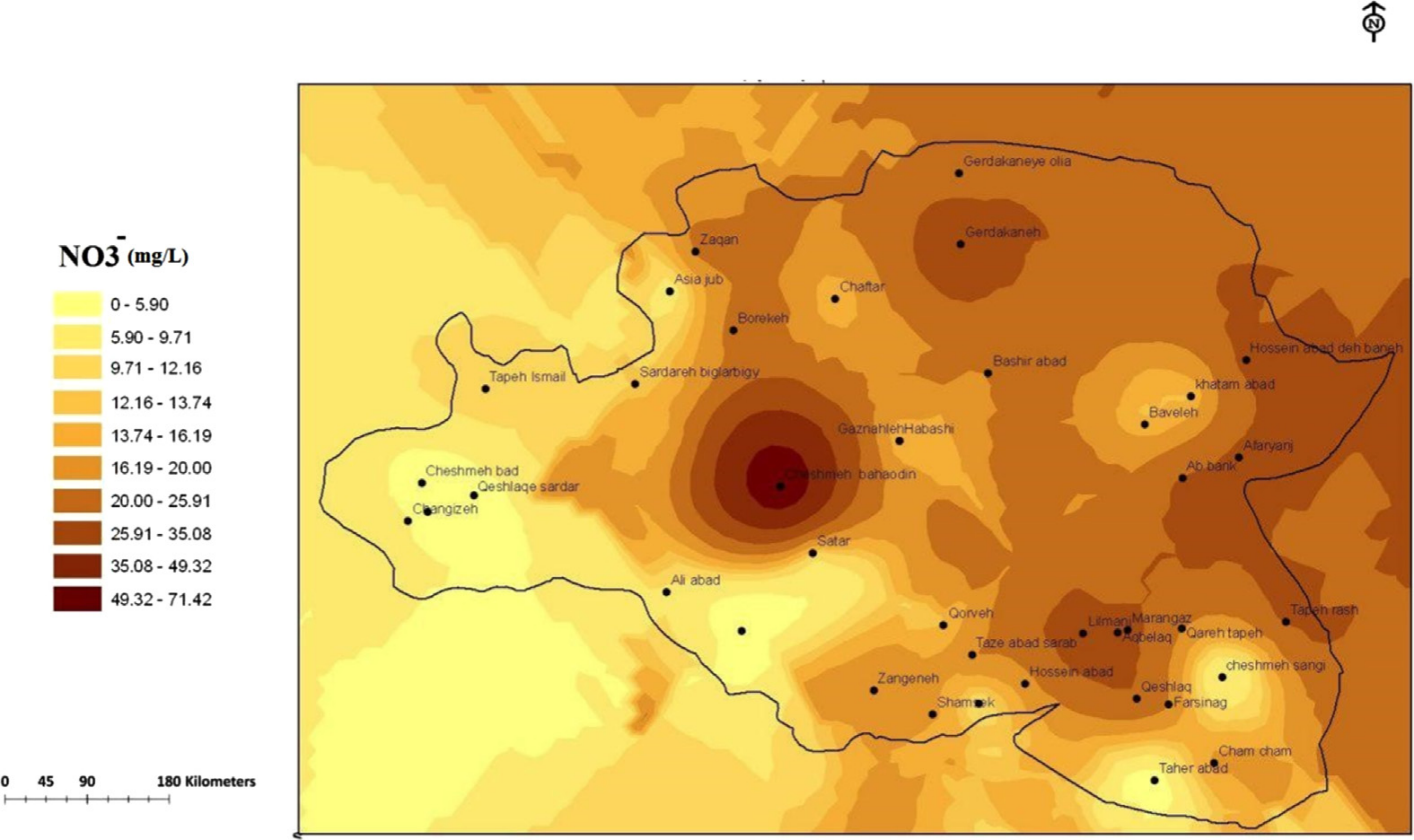
The trend of nitrate (mg/L) changes in water supply sources in the villages of Sonqor, Sampling of low water season, October 2017.

**Fig. 4:**
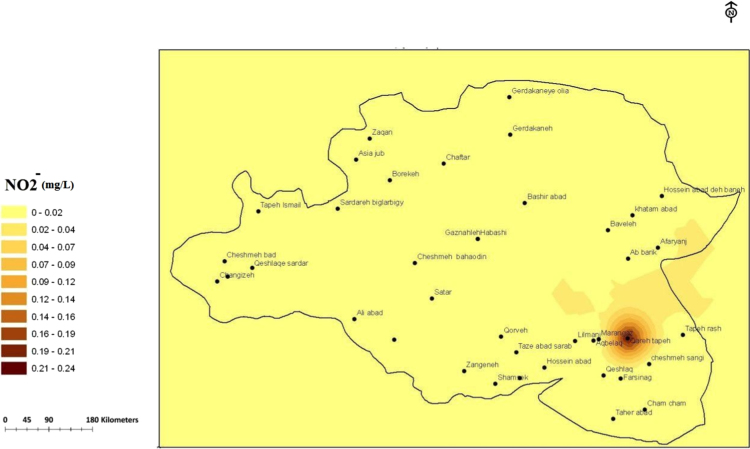
The trend of nitrite (mg/L) changes in water supply sources in the villages of Sonqor, sampling of low water season, October 2017.

Concentration of studied Nitrate–Nitrite in the groundwater of the Sonqor region are summarized in Table 1, Table 2, Table 3, Table 4 and mapping GIS in Fig. 1, Fig. 2, Fig. 3, Fig. 4.

**Table 1:** Mean, maximum and minimum concentration results of the analysis of nitrate and nitrite in high water season, March 2016 from groundwater resources of villages in Sonqor plain, October 2017.

Source	High water season					Low water season				
	Number	Min	Max	Mean	SD	Number	Min	Max	Mean	SD
Qanat	3	5.74	17.68	13.45	6.68	3	17.82	28.89	22.83	5.61
Spring	15	3.94	33.2	12.09	8.92	13	3.09	32.8	12.91	8.62
Well	19	8.66	88.5	24.51	16.74	20	8.56	71.42	23.21	13.36
Total	37	3.94	88.5	18.58	14.55	36	3.09	71.42	19.46	12.22
*P*-value	0.034					0.049

**Table 2:** Mean, maximum and minimum concentration results of the analysis of nitrate and nitrite in low water season, October 2017, from groundwater resources of villages in Sonqor plain.

Parameter	**Unit**	**The range of changes**		**Mean**	**Maximum permissible**	**Maximum desirable**
		**Minimum**	**Maximum**			
Nitrite	mg/L	0	0.1	0.003	–	3
Nitrate	mg/L	3.94	88.5	18.75	–	50

**Table 3:** Comparison of average nitrate concentration in groundwater resources of the villages of the city of Sonqor plain in two high and low water seasons, by source of water supply.

Parameter	**Unit**	**The range of changes**		**Mean**	**Maximum permissible**	**Maximum desirable**
		**Minimum**	**Maximum**			
Nitrite	mg/L	0	0.24	0.006	–	3
Nitrate	mg/L	3.09	71.42	19	–	50

**Table 4:** Comparison of average nitrate concentration in groundwater resources in villages of Sonqor plain in water supply sources in two seasons.

Source	Number	High water season		Low water season		Difference		*P*-value
		SD	Mean	SD	Mean	SD	Mean	
Qanat	3	6.68	13.45	5.61	22.83	12.03	9.38	0.309
Spring	12	9.31	13.7	9	13	4.154	-0.696	0.573
Well	19	16.74	24.51	13.26	23.99	6.141	-0.514	0.72
Total	34	12.35	20.01	14.64	19.72	6.587	-0.295	0.796

## 2 Experimental design, materials and methods

### 2.1 Study area description

**Fig. 5:**
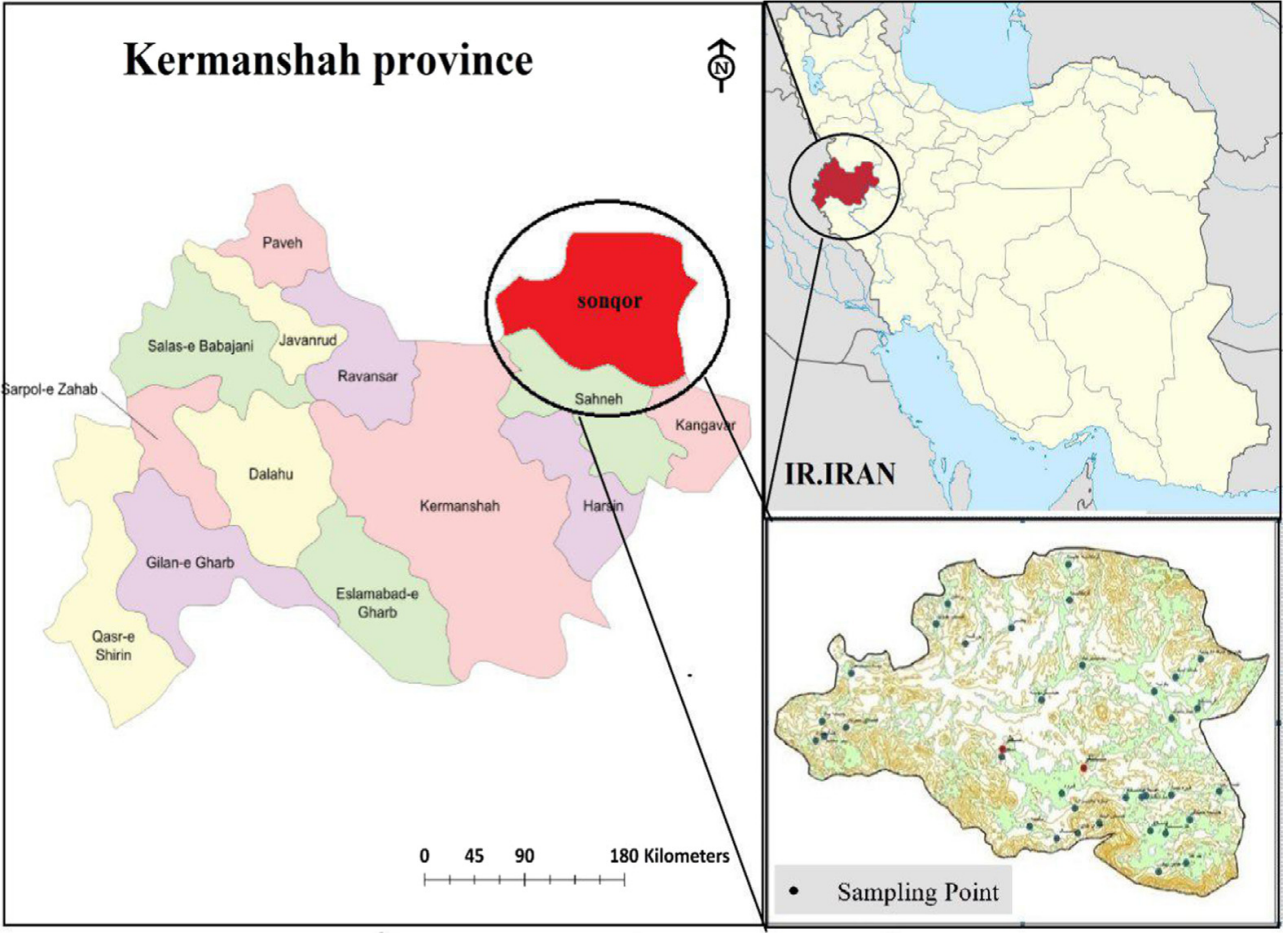
Location of water sampling sites in villages of Sonqor, Kermanshah province.

Sonqor is located at 85 km distance, east north of Kermanshah and 1700 m above sea level [1]. The city׳s population is 51 thousand and contains two districts, two cities, eight rural district and 239 countries. Among countries, 220 ones are haunted and the rest is not inhabited. High level above sea and latitude, have leaded to cold weather formation. The most annual average temperature goes around 17.8 and lowest one is 3.5 °C. The average annual precipitation in Jamishan Dam Lake is approximately 441 mL, with highest precipitation rate on March (79.6 mL) and lowest rate on September month (0.1 mL) are estimated (Fig. 5).

### 2.2 Sample collection and analytical procedures

To evaluate quality of groundwater resources in villages of Sonqor plain, after water resource exploration as the matter of nitrate and nitrite ions, Identification of whereabouts of each groundwater resources which constitute the main potable water resource of villages, Considering the direct relation of nitrate concentration to amount of usage nitrogen containing fertilizer and different irrigation and precipitation in both low and high rate seasons, sampling was conducted in all water ground resource congaing 37 villages resources governed by rural water and waste water organization of Sonqor plain in two times periods. First, March 2016, as the high rate precipitation period and the next period was October 2017 as low rate precipitation. The sampling was carried out in similar areas within one year. Nitrate and nitrite concentrations were determined using spectrophotometry and compared with internal standards [2], [3], [4], [5], [6], [7], [8], [9], [10], [11].

To so sampling, polyethylene bottles with 1-L volume were used and samples were transferred to lab. The whole sampling steps, transferring and data analysis were conducted according to standard method of nitrite and nitrate quantification, exploiting DR5000 Spectrophotometer [12], [13], [14], [15], [16], [17], [18], [19], [20]. Finally, acquired results were compared with internal standards (Institute of Standards and Industrial Research of Iran. No: 1053) [2], [21], [22], [23], [24], [25]. In the proses, three fountains of Aliabad villages got dried out, the sampling was carried out in low rate rain period. The acquired raw data was analyzed by GIS software and after processing, color zoning was prepared using GIS software version 9.3. The criteria that were analyzed by GIS software are including; topography, farms applications, geology, agrology, hydrology and quality of regional water resources.
