# Corrigendum to data on nitrate–nitrite pollution in the groundwater resources a Sonqor plain in Iran [Data in Brief Volume 20 (2018) Pages 394–401]

**DOI:** 10.1016/j.dib.2022.108529

**Published:** 2022-08-05

**Authors:** Davoud Jalili, Majid RadFard, Hamed Soleimani, Samira Nabavi, Hesam Akbari, Hamed Akbari, Ali Kavosi, Abbas Abasnia, Amir Adibzadeh

**Affiliations:** a Department of Environmental Health Engineering, School of Health, Ahvaz Jundishapur University of Medical Sciences, Ahvaz, Iran; b Shahrekord University of Medical Sciences, Shahrekord, Iran; c Nursing Research Center, Golestan University of Medical Sciences, Gorgan, Iran; d Department of Environmental Health Engineering, School of Public Health, Tehran University of Medical Sciences, Tehran, Iran; e Health Research Center, Life Style Institute, Baqiyatallah University of Medical Sciences, Tehran, Iran

The authors regret to say that they have forgotten to cite a key reference for the Table 4 and Fig. 7 during the preparation of draft manuscript. In this regard, the following citation should be considered as the reference for the mentioned sections:

Setareh Parasto, Rezaei Mansour, Hassani Amir Hossam, Zeinatizadeh Ali Akbar. Distribution of groundwater pollution to nitrate in GIS environment: A case study of Songhar plain. Journal of Kermanshah University of Medical Sciences (improvement) (journal of Kermanshah University of Medical Sciences). 1393 [cited 2021October28]; 18 (3 (76)): 157–164. Available from: https://www.sid.ir/fa/journal/ViewPaper.aspx?id=224048.

The authors would like to apologize for the authors of the above-detailed article any inconvenience caused.

